# Dopamine, activation of ingestion and evaluation of response efficacy: a focus on the within-session time-course of licking burst number

**DOI:** 10.1007/s00213-024-06600-1

**Published:** 2024-05-04

**Authors:** Paolo S. D’Aquila

**Affiliations:** Dipartimento di Scienze Biomediche, Università di Sassari, Viale S. Pietro 43/b, Sassari, 07100 Italy

**Keywords:** Behavioural activation, Burst number, Dopamine, Ingestion, Licking microstructure, Reward evaluation

## Abstract

**Rationale:**

Evidence on the effect of dopamine D1-like and D2-like receptor antagonists on licking microstructure and the forced swimming response led us to suggest that (i) dopamine on D1-like receptors plays a role in activating reward-directed responses and (ii) the level of response activation is reboosted based on a process of evaluation of response efficacy requiring dopamine on D2-like receptors. A main piece of evidence in support of this hypothesis is the observation that the dopamine D2-like receptor antagonist raclopride induces a within-session decrement of burst number occurring after the contact with the reward. The few published studies with a detailed analysis of the time-course of this measure were conducted in our laboratory.

**Objectives:**

The aim of this review is to recapitulate and discuss the evidence in support of the analysis of the within-session burst number as a behavioural substrate for the study of the mechanisms governing ingestion, behavioural activation and the related evaluation processes, and its relevance in the analysis of drug effects on ingestion.

**Conclusions:**

The evidence gathered so far suggests that the analysis of the within-session time-course of burst number provides an important behavioural substrate for the study of the mechanisms governing ingestion, behavioural activation and the related evaluation processes, and might provide decisive evidence in the analysis of the effects of drugs on ingestion. However, further evidence from independent sources is necessary to validate the use and the proposed interpretation of this measure.

## Introduction

Rats consuming fluids exhibit licking patterns characterized by discrete sequences of licks – licking ‘bursts’, also referred to as ‘bouts’ or ‘clusters’ – occurring at a rate of approximately 5 to 7 licks per second (Davis 1989, 1996, 2004). The magnitude of such units, referred to as ‘burst size’, is determined by the number of licks per burst and is especially influenced by stimuli related to the orosensory interaction with the reward, such as taste cues. In particular, studies have shown a direct correlation between this measure and the concentration of various sugars. On the other hand, their number in an experimental session, i.e. the ‘burst number’, indicates how many times the subjects initiate “bouts of ingestion” and is influenced also by stimuli unrelated to orosensory contact with the reward, such as post-ingestive cues (D’Aquila and Galistu 2017; Davis and Smith 1992; Dwyer 2012; Johnson 2018a; Sclafani 2001; Smith 2001; Spector et al. 1998). Based upon these observations, the number and size of licking bursts might be regarded, respectively, as indicators of (i) the activation process of a reward-oriented response and (ii) an evaluation process occurring during the consummatory transaction with the reward, reflecting palatability and possibly related to the experience of pleasure (D’Aquila & Galistu 2017; Davis 1989; Davis and Smith 1992; Dwyer 2012; Higgs and Cooper 1998; Schneider et al. 1990; Smith 2001; Spector et al. 1998). It was occasionally reported that burst size can decrease during the session due to post-ingestional signals (e.g. Spector et al. 1998). The experiments from our lab where this possibility was investigated (D’Aquila 2010; D’Aquila and Galistu 2017, 2020), consistently with the results of Davis and Smith (1992), failed to observe within-session changes of this measure – with the exception of the case of water intake (D’Aquila et al. 2019; Davis et al. 1999).

Sucrose ingestion appears to be regulated by both orosensory (Geary and Smith 1985; Smith 2004; Weingarten and Watson 1982) and post-ingestive (Sclafani and Ackroff 2004; Sclafani and Nissenbaum 1987) mechanisms. Dopamine in the mesolimbic system appears to play a crucial role in licking for sucrose solutions (Smith 2004): sucrose licking results in elevated dopamine levels in the nucleus accumbens, and nomifensine-induced dopamine release in this region enhances this behaviour, an effect blocked by dopamine antagonists (Hajnal and Norgren 2001). Findings from studies on the effect of systemic administration of dopamine D1-like and D2-like receptor antagonists on licking microstructure (Canu et al. 2010; D’Aquila 2010; D’Aquila et al. 2012; Galistu & D’Aquila 2012, 2013; Genn et al. 2003; Higgs and Cooper 2000; Liao and Ko 1995; Schneider et al. 1990) and the forced swimming response (D’Aquila & Galistu 2012, 2019) led us to suggest that (i) dopamine on D1-like receptors plays a role in activating reward-directed responses and (ii) the level of response activation is updated – or reboosted – based on a process of evaluation of response efficacy requiring dopamine on D2-like receptors.

A main piece of evidence in support of this hypothesis was the observation that the administration of the dopamine D2-like receptor antagonist raclopride induced a within-session decrement of burst number occurring after the contact with the reward, which we interpreted as an extinction-like effect, i.e. an effect mimicking the effect of reward devaluation on operant responding. It should be stressed that the few published studies focusing on a detailed analysis of the within-session burst number time-course were conducted in our laboratory (D’Aquila 2010, 2020; D’Aquila and Galistu 2017, 2021; D’Aquila et al. 2019; Galistu and D’Aquila 2013, 2020; Galistu et al. 2011). As we shall see, a few pieces of evidence from other laboratories might provide support to the interpretative framework suggested here about the functional meaning of this parameter (Frisina and Sclafani 2002; Robles and Johnson 2017).

The aim of this narrative review is to recapitulate and discuss the evidence in support of the analysis of the within-session burst number time-course as a behavioural substrate for the study of the mechanisms governing ingestion, behavioural activation and the related evaluation processes, and its relevance in the understanding of drug effects on ingestion.

Finally, a note of caution about the interpretation of the data on burst number, which, as reported above, is a measure under the influence of post-ingestive feedback: In several experiments examining the within-session time-course of this measure, drugs were employed that may either activate or inhibit neurotransmitter receptors in the gastrointestinal tract – dopamine antagonists and imipramine (Neuhuber & Wörl 2018; Thomasi and Gulbransen 2023), memantine (Filpa et al. 2016), cannabinoid agents (Camilleri and Zheng 2023). Therefore, it should be born in mind that these pharmacological actions might contribute to the described effects.

## Analytical issues

In our studies, the functional unit upon which all the behavioural analyses were built upon – referred to as licking ‘burst’ or ‘bout’ – was defined as a series of licks with pauses no longer than 400 ms. The choice of this temporal threshold – the pause criterion – may have significant consequences in determining the experimental results, not to mention the possibility to make meaningful comparisons with data from other laboratories. Therefore, since all the studies examining the within-session time-course of burst number were performed in the same laboratory, it is important to discuss with some detail the implications of this choice.

Davis and Smith (1992) identified three regions of distribution of the inter-lick intervals (ILIs) (*verbatim*): “(a) the within-burst distribution (ILIs ≤ 250 ms), (b) the inter-burst interval (IBI) distribution (ILIs > 250 and ≤ 500 ms), and (c) the intercluster interval (ICI) distribution (ILI > 500 ms).” The ILIs relative to the within-burst distribution region reflect the rhythm of the tongue movements and depend upon the activity of a brainstem Central Pattern Generator (CPG). The activity of the CPG can occur independently of proprioceptive feedback and of descending input from the cortex (Moore et al. 2014). Runs of licking bursts separated by an ILI falling in the distribution region > 250 and ≤ 500 ms (IBI), corresponding roughly to one period of the tongue extension-retraction cycle, were termed ‘clusters’ (i.e. clusters of licking bursts). The ‘clusters’ are the functional units of the analysis of licking microstructure, since their size and their number within a session respond in a distinctive way to experimental manipulations involving either orosensory stimulation or post-ingestive cues (see Introduction). Based on the distribution frequencies reported above, the pause criterion for the definition of clusters was set at 500 ms (Davis and Smith 1992).

In our studies, as reported above, we defined such units as ‘bouts’ (e.g. D’Aquila 2010, following Higgs and Cooper 1998) or ‘bursts’ (e.g. D’Aquila and Galistu 2017, following Spector et al. 1998), defined by a pause criterion of > 400 ms (see Higgs and Cooper 1998). Such criterion was selected because this time interval was just longer than the break-point in a log survivor plot of ILIs (see Higgs and Cooper 1998). This value (400 ms) is more than twice the average within-burst ILI (150 ms), is longer than the average IBI (300 ms) and is close to the intercluster interval (500 ms) according to the definitions of Davis and Smith (1992). Importantly, the frequency of ILIs > 400 ms < 500 ms, which might possibly give rise to inconsistencies between the results obtained using these two different pause criteria, appears to be very low (see Fig. 3 in Davis and Smith 1992). Therefore, the definition of licking ‘burst’/‘bout’ in our studies (and in almost all the studies performed in rats by the research groups of Cooper and Higgs) is functionally similar to the ‘cluster’ as defined by Davis and Smith (1992). However, caution should be exerted in interpreting the data with relevant effects on the within-burst lick frequencies.

In an influential study, Spector and colleagues (1998) examined the effects on licking microstructure of the manipulation of sucrose concentration and feeding as a function of the pause criterion (comparing the durations of 0.3, 1, 3, 10, 30 and 100 s). The results of this study provide support to the choice of a 1 s pause criterion and were consistent to those obtained by Davis and Smith using the 500 ms pause criterion (e.g. Davis and Smith 1992). Prompted by this study, Higgs and colleagues (Higgs et al. 2003) analysed a set of data comparing the 1 s *versus* the 400 ms pause criterion, with the differences between the results of the two analyses being negligible.

## The analysis of the within-session time-course of burst number suggests a role for dopamine D2-like receptors in the ‘reboosting’ of ingestion activation

### About the possible role of dopamine in hedonic impact and behavioural activation

The demonstration that dopamine receptor antagonists diminish instrumental responses for food and other rewards – thus mimicking the effect of reward devaluation (“extinction-mimicry”) – provided the main piece of evidence in support of the so-called anhedonia-hypothesis, which posits a role for dopamine in hedonic impact (Fouriezos et al. 1978; Wise 2008; Wise et al. 1978). Extinction-mimicry with low doses of dopamine antagonists has also been reported in rats navigating an alley for food (Chausmer and Ettenberg 1997; Ettenberg and Camp 1986a, b; McFarland and Ettenberg 1998) or heroin (McFarland and Ettenberg 1995), as well as in studies measuring freely delivered food consumption (Wise 2004).

However, successive findings have significantly challenged this view. Indeed, lesioning dopamine mesolimbic ascending pathways, though nearly completely eliminating reward-oriented responses, does not affect appetitive taste reactions to sucrose, which are considered as hedonic responses (Berridge et al. 1989). This led to the suggestion that dopamine, rather than being linked to ‘liking’, i.e. hedonic impact, is involved in ‘wanting’, i.e. the attribution of incentive salience to reward-associated stimuli. This process would consist in the transformation of the neural representation of a reward-related stimulus into a motivationally potent incentive, thus acting as a trigger for the activation of the appropriate reward-directed response (Berridge 2007; Nguyen et al. 2021; Olney et al. 2018; Warlow and Berridge 2021). Moreover, rats with dopamine depletion in the nucleus accumbens tend to shift their responses toward less effortful choices but still maintain the ability to choose a larger reward when no additional effort is required, suggesting a role for dopamine in response effort allocation but not in the ability to assess the reward-value (Salamone 2007; Salamone et al. 2005, 2007; Salamone and Correa 2024; Treadway and Salamone 2022). Other theoretical perspectives posit an involvement of dopamine in cost-benefit computational mechanisms (Baldo and Kelley 2007; Cannon and Palmiter 2003; Hori et al. 2021; Kroemer et al. 2016; Niv et al. 2005; Phillips et al. 2007).

The common aspect of these accounts is the recognition of the involvement of dopamine in the *activation* of reward-oriented responses, while ruling out (or simply disregarding) its involvement in hedonic impact (Robbins and Everitt 2007). To reconcile the evidence of extinction mimicry with the incentive salience attribution hypothesis, it was suggested the concept of ‘reboosting’, a process by which the contact with the reward serves to update (or reboost) the level of incentive salience attributed to its associated stimuli (Berridge 2007). This, however, ascribes to dopamine a role in some evaluation process involving a contact with the reward.

### Discrepancies between licking microstructure and taste reactivity studies about the possible role of dopamine in hedonic impact

Another apparent inconsistency in regard to the possible role of dopamine in hedonic impact arises from the dissociation between evidence obtained studying licking microstructure and taste reactivity (see Dwyer 2012 for a detailed account). Indeed, at variance with the results of studies on taste reactivity to intraoral infusion of sweet solutions, treatment with dopamine antagonists results in an effect suggesting a blunted hedonic response, namely a reduction of the size of licking bursts. In particular, it was shown that the effect of the dopamine D2-like receptor antagonist raclopride induced on this measure an effect similar to that exerted by sucrose dilution, which is an instance of reward devaluation (Schneider et al. 1990). While it was demonstrated that dopamine D2-like receptor blockade reduced burst size (Genn et al. 2003; Higgs and Cooper 2000; Liao and Ko 1995; Schneider et al. 1990), less clear was the evidence on the effects of dopamine-D1 like receptor blockade (Schneider 1989; Schneider et al. 1989a; but see Liao and Ko 1995; Schneider et al. 1989b). It might be also worth noting here that earlier studies failed to observe extinction-mimicry in licking, with the measure investigated being the within-session time-course of lick number (Gramling et al. 1984; Gramling and Fowler 1986).

### The response to dopamine D1-like and D2-like receptor antagonists of the within-session time-course of burst number

To further explore the role of dopamine and of dopamine receptor subtypes on ingestion activation and on reward evaluation, we performed a study comparing the effect of the dopamine D1-like receptor antagonist SCH 23390 and of the dopamine D2-like receptor antagonist raclopride on the microstructure of licking for a 10% sucrose solution. In this study, we examined for the first time the effect of dopamine antagonists on the within-session time-course of burst number (D’Aquila 2010). The results align with prior research demonstrating that D2-like receptor antagonism can diminish sucrose consumption by reducing the size of licking bursts. Most importantly in relation to the scope of this review, a clear distinction between the effects of dopamine D1-like and D2-like receptor antagonists was revealed by the analysis of the within-session time-course of burst number.

As previously observed with reward devaluation or neuroleptic administration in instrumental responding (Wise 2008; Wise et al. 1978), the effect of raclopride on burst number took place only after a few minutes from the beginning of the experimental session and led to either a compensatory increase (at low doses) or a decrease (at high doses) in this measure. Notably, raclopride, across all examined doses, did not influence the latency to the first lick. These observations rule out a role for D2-like receptors in the direct activation of the licking response but suggest their involvement in a process of reward evaluation/hedonic impact.

In contrast, the D1-like receptor antagonist SCH 23390 reduced licking exclusively by diminishing the number of bursts. This was demonstrated by the overlapping pattern of the time-course curves for lick number and burst number, while no effects were observed on burst size. As previously observed with the same drug in operant responding (Sanger 1987), the effect was present since the beginning of the session. These findings suggest a role for dopamine D1-like receptors in the activation of the licking response. Supporting this interpretation, in rats engaged in daily alley running sessions for food, the dopamine D1-like receptor antagonist SCH 39166, unlike raclopride, failed to prevent reinstatement when administered during a reinforced trial amid extinction (Chausmer and Ettenberg 1997). Conversely, administration of dopamine D2-like, but not D1-like, receptor agonists reinstated operant responding for cocaine (Self et al. 1996), while mice lacking dopamine D1 receptors showed reduced instrumental responding for sucrose but exhibited normal preference when sucrose was available in a free-choice paradigm (El-Ghundi et al. 2003).

A study investigating the contributions of Pavlovian incentive motivation to cue potentiated feeding showed that licking bouts can be elicited by food-paired cues and that this effect depends on dopamine D1 receptor activation (Marshall et al. 2018). This result is consistent with our suggestion that the responses to the reward-associated cues depends on dopamine D1-like receptor stimulation (D’Aquila 2010). However, at variance with this interpretation, it was reported that the ability of a conditioned stimulus to elicit approach behaviour can be dissociated from its ability to initiate bursts of licking (Johnson 2018b).

The same results – and in particular the same response patterns of the within-session time-course of burst number – were obtained in a successive study examining the effect of the dopamine-D1 and -D2 like antagonists on the microstructure of licking for a NaCl solution (Galistu and D’Aquila 2013).

In a successive study examining the effect of the same antagonists on licking for water, the observed effect of dopamine D1-like receptor blockade was in keeping with the results described above, but we failed to observe the extinction-like pattern of the within-session time-course of burst number with the administration of the dopamine D2-like receptor antagonist (D’Aquila et al. 2019). This apparent inconsistency might be explained by the observation that water drinking both in normal conditions and after water deprivation – in contrast to the appetitive intake of NaCl and sugars – can occur even in the absence of taste signals (Zocchi et al. 2017). Moreover, water exerts a strong postingestional inhibitory effect on burst size (Davis et al. 1999), which blurs the distinction between the effects of post-ingestive and orosensory stimuli. These observations suggest that the reboosting of the licking response to water, at variance with the case of sucrose and NaCl, does not depend on palatability.

These data support the view that dopamine on D2-like receptors is involved in reward evaluation/hedonic impact, while dopamine on D1-like receptors is involved in the activation of reward-oriented responses. Moreover, the similarity between the within-session time-course of burst number and instrumental responding for a reward in response to dopamine D1-like and D2-like antagonists supports the suggestion that the emission of licking bursts and of operant responses might depend on common neural substrates (Galistu and D’Aquila 2013). Most importantly in relation to the aim of this review, these results show that the analysis of the within-session time-course of burst number provided a fundamental piece of evidence to interpret the different effect of dopamine D1-like and D2-like receptor antagonists on ingestion in functional terms.

## The effect of dopamine antagonists on the forced swimming response suggests the involvement of dopamine D2-like receptors in response efficacy evaluation

Within the interpretative framework suggested above, we proposed “that the level of activation of the responses (or the incentive salience attribution) to the reward associated cues be updated, or reboosted, on the basis of the dopamine D2-like receptor-mediated ‘contingent’ reward evaluation occurring during the consummatory transaction with the reward”. The contingent value of the reward might be regarded as the computational term which provides the basis to determine the level of activation of the reward-directed response, hence the cost of the response – in terms of effort – which is congruous in the given physiological, psychological and environmental condition (D’Aquila 2010). This account is consistent with the theoretical view which regards dopamine’s role in terms of cost-benefit analysis based choice and response effort allocation (Salamone et al. 2005, 2007, 2009; Salamone and Correa 2024; Treadway and Salamone 2022). However, the experimental measures provided by the study of licking microstructure are not particularly apt to be interpreted in terms of cost-benefit analysis, since the effort demand of licking behaviour is very low.

Thus, we decided to test the proposed hypothesis in the forced swimming test (D’Aquila and Galistu 2012). Indeed, this paradigm can be interpreted in terms of goal directed behaviour (with the goal being escape/survival), elicits an effortful response, and shows an update of the behavioural response both within sessions and between sessions. Typically, the subjects (rats or mice) are placed in cylinders filled with water from which escaping is impossible. They are kept in this condition for 15 min, rescued, and a second session lasting 5 min is performed 24 h later. The subjects exhibit a vigorous response aimed at escape, consisting in attempts to climb the cylinder wall (climbing), swimming and diving. The level of activity declines in the course of the session. In the second session performed 24 h later a reduced level of activity is observed. Antidepressants, administered between the two sessions, reduce immobility in the second session (Cryan et al. 2005; de Kloet and Molendijk 2016; Molendijk and de Kloet 2019; Porsolt et al. 1977, 1978a, b). Dopamine D1-like and D2-like receptor antagonists prevent the effect of antidepressants (Asakura et al. 1994; Baamonde et al. 1992; Borsini et al. 1988; D’Aquila et al. 1994, 2010; Maj et al. 1992a, b; Shimazu et al. 2005; Vaugeois et al. 1996; Yamada et al. 2004). To test the proposed hypothesis in the forced swimming test, where no reward is delivered to the subjects, the evaluation process revealed by the burst size and by the within-session time-course of burst number – which respond to changes in palatability (D’Aquila and Galistu 2017) – might be conceived as “evaluation of response efficacy”. In the case of licking, the efficacy of the response depends on the rewarding value that the brain assigns to the ingested volume, which is related to the intensity of the sweet taste, possibly because sweetness, in natural conditions, is a predictor of readily available caloric content (see Beauchamp 2016 for a critical discussion on this point). This concept can be aptly applied to the case of the forced swimming test, where the subjects reduce the activation of their escape attempts, both within sessions and between sessions, after perceiving that their effort will not result in a successful outcome, i.e. after a negative evaluation of the efficacy of their costly behavioural response.

The subjects treated with the dopamine D1-like receptor antagonist SCH 23390 showed a reduced climbing since the beginning of the session, with the activity declining within the session as observed in the control group. According to the proposed interpretative framework, dopamine D1-like receptor blockade reduced the level of behavioural activation, but the process of evaluation of response efficacy responsible of the suppression of the unsuccessful behavioural response was spared. The possibility that the reduced climbing observed after treatment with SCH 23390 might depend on motor effects must be taken into account (see next section and D’Aquila & Galistu 2012 for a discussion on this point).

In contrast, the subjects treated with the dopamine D2-like receptor antagonist raclopride failed to show a substantial decline of climbing within the session. In the framework of the proposed hypothesis, this behaviour might reveal an impairment of the process of evaluation of response efficacy, leading to persisting in a costly but ineffective response. These results were confirmed in a successive study from our lab (D’Aquila and Galistu 2019). (Incidentally, only a fine-grained analysis of the within-session response time-course led to the distinction between dopamine D2-like receptor blockade *versus* imipramine effects in the forced swimming test in the latter study.)

Thus, the study of the effect of dopamine antagonists on the forced swimming response provide support to a generalization of the proposed hypothesis, positing that dopamine D1-like receptors are involved in the activation of goal directed responses, and that the level of activation is updated, or reboosted, on the basis of the evaluation of response efficacy. The subjects would evaluate response efficacy taking into account the value of the goal (which is the rewarding value of the solution for licking and survival in the case of the forced swimming condition) and the distance from the goal, i.e. the estimated additional effort required to reach the goal. If the distance from the goal is sufficiently small, as it happens with a slight reward devaluation, the ineffective response can be corrected with an increase in response activation. If the distance from the goal cannot be overcome by increasing response activation, as it happens with drastic reward devaluation or in the forced swimming condition, the response is extinguished. This account would explain the effects both of the small and of the high doses of dopamine D2-like receptor antagonists, which result, respectively, in increases or decreases of the response rate in the case of instrumental responding, and of the burst number in the case of licking.

## Motor *versus* motivational processes and behavioural activation

As we have seen, the notion that the within-session decline induced by dopamine antagonists in operant responding mimics the effects of reward devaluation played a crucial role in supporting a role for dopamine in mediating hedonic impact (Wise 2004, 2008; Wise et al. 1978). However, several studies have identified significant differences between extinction and the effects of dopamine antagonists, casting doubt on their functional equivalence and suggesting alternative explanations in terms of motoric effects (Rick et al. 2006; Salamone 1986; Sanger 1986; Spivak and Amit 1986; Tombaugh et al. 1980, 1982). Of particular relevance is the evidence of within-session impairments of motor competence in response to antipsychotics, such as increased operant response duration in rats (Liao and Fowler 1990) and micrographia with an extinction-like pattern in humans (Haase and Janssen 1985). Thus, a significant challenge in interpreting the impact of dopamine antagonists as ‘motivational’ lies in the possibility that a simpler explanation could be attributed to impaired motor function.

Early studies on the effects of administration of dopamine antagonists on licking have shown evidence of specific motor effects, with reduced lick number accompanied by decreased force of tongue protrusion, increase of the duration of individual licks and of the inter-lick intervals (Fowler and Mortell 1992; Gramling et al. 1984; Gramling and Fowler 1986). The combination of these effects can be revealed by the reduction of the intra-burst lick rate, defined as the within-burst frequency of licking (i.e. the reciprocal of the within-burst average ILI). Therefore, the intra-burst lick rate is regarded as a sign of motor impairment. The dose-ranges of SCH 23390 and raclopride used in all studies from our laboratory can be considered as borderline in regard of the ability to reduce the intra-burst lick rate. Indeed, in some studies we failed to observe significant effects on the intra-burst lick rate (SCH 23390: D’Aquila et al. 2012; Galistu & D’Aquila 2012, 2013; Raclopride: Galistu and D’Aquila 2013; D’Aquila et al. 2019; but see Canu et al. 2010; D’Aquila 2010d Aquila et al. 2012; with raclopride, but not SCH 23390, reducing the intra-burst lick rate). Relevant effects on the other measures, especially burst number for SCH 23390 and burst size for raclopride, were observed even at doses which did not affect the intra-burst lick rate (e.g. D’Aquila 2010). With raclopride, *increased* burst number was observed at doses *reducing* the intra-burst lick rate (e.g. Canu et al. 2010). Thus, neither the effects of these two dopamine antagonists on the motivationally relevant measures of licking microstructure, nor the observed differences between their effects on these measures, can be straightforwardly accounted for in terms of motor function impairment. It is relevant in this context to recall that both SCH 23390 and raclopride failed to affect lick efficiency at doses which resulted in reduced intra-burst lick rate (D’Aquila 2010).

Given that (i) motivation can be operationally defined as the likelihood to emit a goal-directed motor output, and (ii) dopamine is involved both in motivation and in motor control, attempts to distinguish between motor *versus* motivational effects of dopamine antagonists involve the risk of circularity (see Salamone et al. 2005, for a discussion on this point). Indeed, Parkinson’s disease – the movement disorder caused by the loss of mesencephalic dopamine neurons – can result in appetitive motivational deficits (Shore et al. 2011), and patients with neuroleptic-induced Parkinsonism show micrographia with an extinction-like pattern (Haase and Janssen 1985). Along this line of reasoning, it was suggested that “an impaired motivational background might be the basis for the existence of slowness of movement in dopamine deficiency conditions” (Keitz et al. 2003). In a study reporting the comparison between the effects of a dopamine antagonist and extinction on instrumental responding, the author refers to “incentive-related motor activity” (Salamone 1986). A possible way out might be provided by the concept of ‘behavioural activation’, which covers this “area of overlap between motivation and motor function” (see Salamone et al. 1997, 2007).

Notably, both the concept of ‘activation’ and of ‘evaluation of response efficacy’, which might account for the differences between the effect of dopamine D1-like and D2-like receptor blockade in ingestive behaviour and in other paradigms – such as the forced swimming response (D’Aquila and Galistu 2012, 2019) and paradigms investigating the reinforcing properties of food (Chausmer & Ettemberg 1997; Ettenberg and Camp 1986b; McFarland & Ettemberg 1998), water (Ettenberg and Camp 1986a) and addictive drugs (McFarland & Ettemberg 1995) – can fit either a ‘motivational’ or a ‘motor’ interpretative framework.

### Effect of changes in sucrose concentration on the within-session time-course of burst number

The interpretation of the effect of dopamine-D2 like receptor blockade as an extinction-like effect requires that the same response pattern be observed exposing the subjects to reward-devaluation. To test this prediction, rats with daily exposure to a 10% (high) sucrose concentration solution were offered, as a devalued reward, a 2% (low) sucrose solution in the test days (D’Aquila and Galistu 2017). The results of this experiment confirmed the previous finding that sucrose dilution results in a reduced burst size (see Johnson 2018a). Most importantly, they confirmed the prediction about the within-session time-course of burst number. Interestingly, while in the first devaluation session we observed a drop in burst number after a few minutes of contact with the reward, i.e. the response pattern characterizing the process of response extinction, after one or two more devaluation sessions the drop of burst number was anticipated to the beginning of the session. This might suggest a learning process leading to a more rapid update of the level of activation of the emission of licking bursts in response to reward devaluation.

In a parallel experiment reported in the same paper, we compared the response to daily exposure either to a high or to a low sucrose concentration solution in two separate groups of rats, i.e. in a condition with a difference in the reward value (2% *versus* 10% sucrose concentration), but not involving reward-devaluation. A difference in whole-session lick number – i.e. a difference in ingestion levels – emerged since the first session in favour of the high concentration group. This difference was due to increased burst number in the first four sessions and in differences in burst size in the successive sessions. Notably, in the sessions showing significant differences in whole-session burst number, the within-session time-course of this measure differed between the two groups since the beginning of the session – with a lower level in the low concentration group –, as observed with dopamine-D1 like receptor blockade in licking (D’Aquila 2010; D’Aquila et al. 2019; Galistu and D’Aquila 2013) and in instrumental responding (Sanger 1987). This observation shows also that the measure of burst number responds more promptly than burst size to the reinforcing value of the reward.

The high concentration group was also exposed to sessions of reward devaluation, while the low concentration group was exposed to an upshift in sucrose concentration in the conclusive session of the experiment. The results of the devaluation sessions replicated both the extinction-like response pattern of the burst-number time-course in the first devaluation session and the anticipated drop in burst number since the beginning of the session in the successive tests. Notably, in the first devaluation session, i.e. the one showing the extinction-like pattern in the burst number time-course, we failed to observe a statistically significant reduction of burst size. This observation suggests that the within-session decrement of burst number might be a more reliable sign of reduced hedonic impact than the reduced burst size and might have important implications in the interpretation of drug effects on licking microstructure (see below *Evidence from other laboratories*).

Thus, in light of these findings, one can affirm that dopamine D2-like receptor blockade mimics the effects of reward devaluation, while dopamine D1-like receptor blockade reproduces a condition with a lower level of behavioural activation not involving reward devaluation, with the level of behavioural activation being proportional to the reward value. Further studies are warranted to determine whether the similarity between these responses to dopamine antagonists on the one hand, and to manipulations of the reward value on the other hand, depends on common neural substrates.

The increase in burst number observed across sessions was steeper in the high concentration group, reflecting the higher reinforcing value of the high concentration sucrose solution. This difference in the speed of the progressive increase in burst number was paralleled by an almost specular progressive reduction of the latency to the first lick. Notably, latency is considered as a (negative) measure of activation and is influenced both by motor and by motivation mechanisms (Salamone et al. 1997, 2016). This observation provides support to the interpretation of burst number as a measure of behavioural activation.

The upshift in sucrose concentration resulted in an increase in burst number which was present since the beginning of the session, showing an immediate update of response activation to the level which is appropriate for the more valuable reward, in contrast to the case of reward devaluation, with the high response level persisting for several minutes before dropping to a lower level. This difference between the response to a positive or a negative change in the reward value can have an adaptive explanation. Indeed, persisting in a low cost response in the presence of a devalued reward leaves room to the chance to compensate for small reductions of the reward value with an increase in response activation (Wise 2004). However, no advantage can be obtained in delaying the increase in response activation in the presence of a reward of increased value. Consistently with this observation, an increase of burst number at the beginning of the session was also shown in response to clozapine administration, which exerted a prohedonic effect revealed by an increased burst size (Galistu et al. 2011).

Thus, the analysis of the within-session time-course of burst number, but not of burst size, reveals specific activation patterns which differ in the response to reward devaluation – as well as to increased reward value –, with respect to the response to sucrose solutions of different reinforcing values in separate groups of subjects. These response patterns are mimicked by the effect, respectively, of dopamine D2-like and dopamine D1-like receptor blockade (D’Aquila 2010; D’Aquila et al. 2019; Galistu and D’Aquila 2013).

## The relevance of the analysis of the within-session time-course of burst number in the study of drug effects on ingestion

### Blunted hedonic response to sucrose induced by memantine

In a study aimed at the investigation of the motivational effects of the NMDA receptor antagonist memantine on ingestion, we compared the effect of two administration schedules on the microstructure of licking for sucrose. For a week, one group of subjects was treated with memantine 1 h before a 30-min daily session (‘before testing’ group). Another group was treated immediately after each daily session (‘after testing’ group). Behavioural tests were performed also for 15 days after treatment discontinuation (Galistu & D’Aquila 2020).

In the ‘before testing’ group, ingestion was reduced in the first sessions due to reduced burst size, which persisted for the duration of the treatment. However, due to an increase in burst number across sessions, by the end of the treatment, ingestion in the memantine-treated subjects had recovered to the level of the control group. The analysis of the within-session time-course of burst number revealed that memantine-treated subjects exhibited a steeper decline of this measure in the first session, resembling the effect of sucrose dilution (D’Aquila and Galistu 2017), while slight increases were observed within the last sessions, resembling the compensatory response to mild reward devaluation in operant behaviour (Wise 2008; Wise et al. 1978). These observations suggest that before-session memantine administration results in a blunted hedonic response, consistently with the notion that memantine interferes with the hedonic/non-homeostatic mechanisms regulating food intake and food-seeking (Bisaga et al. 2008; Foltin et al. 2008; Popik et al. 2011; Smith et al. 2015). It is worth noting that also the compensatory increase in burst number across sessions resembles the response to mild reward devaluation in instrumental responding (Wise 2004), thus providing further support to the suggestion that a common mechanism might underlay the emission of licking bursts and of operant responses (Galistu and D’Aquila 2013).

Notably, the proposed reading of the burst-number time-course analysis leads to conclusions which are consistent, on the one hand, with the more generally accepted interpretation of reduced burst size as a sign of blunted hedonic response, and, on the other hand, with the views of other research groups based on results from different behavioural paradigms (Bisaga et al. 2008; Foltin et al. 2008; Popik et al. 2011; Smith et al. 2015).

In the ‘after testing’ group, the level of ingestion dropped almost to naught due to a drastic and progressive decrease in burst number across sessions. A slow recovery was observed after treatment discontinuation. Thus, the effect of post-session administration, possibly due to the development of conditioned taste aversion, consisted in a dramatic reduction of behavioural activation. Consistently with the proposed functional interpretation of the within-session time-course of burst number, a reduced level of this measure was present since the beginning of the session, starting from the session following the first treatment. As previously observed (D’Aquila et 2017), the changes across sessions in burst number level are paralleled by specular changes in latency values, providing further support to the interpretation of burst number as a measure of behavioural activation.

### Does imipramine exert a prohedonic effect in sucrose ingestion?

In the course of a chronic treatment with imipramine (D’Aquila and Galistu 2021), an increased burst size was observed with imipramine (10 mg/kg) in sessions run 1 h after a daily treatment, possibly suggesting an increased hedonic response to sucrose. However, this result is inconsistent with the result of a parallel study (D’Aquila and Galistu 2020). Moreover, very few studies in decades reported prohedonic effects in ‘normal’ subjects (Collu et al. 1997; Fibiger and Phillips 1981; Papp 1988) – ‘normal’ as opposed to ‘stressed’, since a great deal of evidence showed the ability of these drugs to reverse stress-induced anhedonia, while no prohedonic effects were observed in the non-stressed control groups (e.g. Ménard et al. 2016; Willner 2017).

The prohedonic interpretation is also inconsistent with the proposed interpretation of the analysis of the within-session time-course of burst number. Indeed, as we have seen, the reaction to an increase in the reward value (D’Aquila and Galistu 2017) or to a putatively prohedonic drug such as clozapine (Galistu et al. 2011) was characterized by an increased burst number at the beginning of the session, along with an increased burst size in the case of clozapine. In contrast, the increased burst size observed in the course of imipramine treatment was paralleled by reduced burst number since the beginning of the session. This response pattern was also observed in rats licking for water treated with the dopamine D1-like receptor antagonist SCH 23390, and the increased burst size was interpreted – at least in part – as a compensatory response to the reduced activation of ingestive behaviour (D’Aquila et al. 2019).

### CB1 receptors and the hedonic impact of sucrose

In a recent study, we examined the effect of the combined administration of the CB1 receptor antagonist-inverse agonist rimonabant and of the non-selective cannabinoid agonist HU-210 on licking for sucrose (D’Aquila 2020). One aim of the study was to attempt to reconcile apparently inconsistent findings from previous studies suggesting either a the role for CB1 receptors in hedonic impact/reward evaluation – supported by effects on burst size (Higgs et al. 2003; Robinson and McCool 2015; Sanchis-Segura et al. 2004) – or, alternatively, in incentive salience attribution/behavioural activation – supported by effects on burst number (Grey et al. 2012; Thornton-Jones et al. 2007). In our study, both treatments failed to affect burst size. The reduced lick number observed with both drugs was due exclusively to reduced burst number. Notably, rimonabant (0.5 mg/kg) reduced burst number late in the session, possibly suggesting a blunted hedonic response, i.e. reduced ‘liking’. In contrast, a higher dose (1 mg/kg) reduced burst number at the beginning of the session, possibly suggesting reduced ‘wanting’ (D’Aquila and Galistu 2017).

Also HU-210 decreased burst number late in the session. However, this effect was accompanied by a reduced intra-burst lick rate, suggesting caution in interpreting this observation as a blunted hedonic response. Indeed, as we have seen, a number of studies suggest that the extinction-like pattern observed after neuroleptic administration – and HU-210 shares with neuroleptics the ability to induce motor impairment (Ottani et al. 2002) – might be explained more parsimoniously as motor impairment rather than as a blunted hedonic response (Rick et al. 2006; Salamone 1986; Sanger 1986; Spivak and Amit 1986; Tombaugh et al. 1980, 1982). Most importantly in relation to the topic discussed here, HU-210 reversed the effect of the high dose of rimonabant, yielding an increase of burst number at the beginning of the session, as previously observed with an upshift in sucrose concentration (D’Aquila and Galistu 2017) and with clozapine (Galistu et al. 2011). This observation suggests that HU-210 reversed the anhedonic effect of rimonabant, on the one hand supporting the interpretation of the effect of rimonabant in terms of reduced hedonic impact, and on the other hand ruling out the possible anhedonic effect of the cannabinoid agonist HU-210. This interpretation – built upon the analysis of the within-session time-course of burst number – is consistent with a great deal of evidence from both human and animal studies on the involvement of CB1 receptors in hedonic responses (e.g. Beyer et al. 2010; Horder et al. 2010; Wang et al. 2010).

### Evidence from other laboratories

To the best of my knowledge, no other laboratory has ever reported a detailed analysis of the burst number within-session time-course with a temporal resolution comparable to that of the studies described in this review (i.e. 3-min time-bins within a 30-min session). (For example, Hartfield et al. 2003 reported the data from a 30-min session divided into three 10-min time-bins.) However, in a set of data showing no effects on burst size, the within-session time-course of burst number can be inferred from the lick number time-course. This is the case of two studies the interpretation of which can be reëxamined in light of the evidence discussed in this paper. In brief, the lack of effect on the initial lick rate and on burst size led the authors to question the ability to blunt the hedonic response (i) of the opioid antagonist naltrexone (Frisina and Sclafani 2002) – an observation at variance with a great deal of experimental evidence (Dum et al. 1983; Kirkham and Cooper 1988a, b; Parker et al. 1992; Rockwood and Reid 1982; Yamamoto et al. 2000) – and (ii) of the dopamine receptor antagonist eticlopride (Robles and Johnson 2017). However, both studies reported a within-session decrement of lick number – hence of burst number – with the response of the treated subjects being similar to that of the control group at the beginning of the session, followed by a steeper decline.

It is important to stress that reduced burst size and initial lick rate were suggested to indicate a reduced hedonic response because these effects can be observed after sucrose dilution, i.e. after reward devaluation (see Johnson 2018a). However, we have shown that reward devaluation might induce a within-session decline of burst number even in absence of reduced burst size and of the initial lick rate (D’Aquila and Galistu 2017). Thus, the evidence presented in these two studies is consistent with the interpretation of the effects of naltrexone and eticlopride as a blunted hedonic response, in spite of the lack of reduced burst size.

Finally, the latter study provides an independent replication of our previous finding on the ability of dopamine D2-like receptor blockade to induce an extinction-like pattern on burst number. Notably, this response pattern was replicated with a different drug (eticlopride *versus* raclopride), a different route of administration (lateral ventricle infusion *versus* systemic administration) and in a different species (mouse *versus* rat) (Robles and Johnson 2017).

## Conclusions

The data from the experiments performed in our laboratory examining the within-session time-course of burst number show a great deal of internal consistency in regard of the behaviour of this measure in response to changes in sucrose concentration and to dopamine D1-like and D2-like antagonist administration. Moreover, these data fit within an interpretative framework that, when used to explain the behaviour of this measure in response to different drugs (clozapine, memantine, imipramine, cannabinoid agents), provides an account showing a great deal of consistency, on the one hand, with the other results from the analysis of licking microstructure within the same experiments, and, on the other hand, with evidence from independent sources not necessarily involving the investigation of licking microstructure. However, these findings were never subjected to further investigation by other laboratories, with the only possible exception of the cited study on the effect of the dopamine D2-like antagonist eticlopride (Robles and Johnson 2017).

In conclusion, the evidence gathered so far suggests that the analysis of the within-session time-course of burst number provides an important behavioural substrate for the study of the mechanisms governing ingestion, behavioural activation and the related evaluation processes, and might provide decisive evidence in the analysis of the effects of drugs on ingestion. However, further evidence from independent sources is necessary to validate the use and the proposed interpretation of this measure.
